# The clinical and cost-effectiveness of brief advice for excessive alcohol consumption among people attending sexual health clinics: a randomised controlled trial

**DOI:** 10.1136/sextrans-2014-051561

**Published:** 2014-06-16

**Authors:** Mike J Crawford, Rahil Sanatinia, Barbara Barrett, Sarah Byford, Madeleine Dean, John Green, Rachael Jones, Baptiste Leurent, Michael J Sweeting, Robin Touquet, Linda Greene, Peter Tyrer, Helen Ward, Anne Lingford-Hughes

**Affiliations:** 1 Centre for Mental Health, Imperial College, London, UK; 2 Centre for the Economics of Mental and Physical Health, King's College London, London, UK; 3 Central and North West London NHS Foundation Trust, London, UK; 4 Chelsea and Westminster Hospital NHS Foundation Trust, London, UK; 5 PRIMENT Clinical Trials Unit, University College, London, UK; 6 MRC Biostatistics Unit, Cambridge, UK; 7 Imperial College Healthcare NHS Trust, London, UK; 8 School of Public Health, Imperial College, London, UK; 9 Centre for Neuropsychopharamacology, Imperial College, London, UK

**Keywords:** Sexual Behaviour, Substance Misuse, Clinical Trials

## Abstract

**Objectives:**

To examine the clinical and cost-effectiveness of brief advice for excessive alcohol consumption among people who attend sexual health clinics.

**Methods:**

Two-arm, parallel group, assessor blind, pragmatic, randomised controlled trial. 802 people aged 19 years or over who attended one of three sexual health clinics and were drinking excessively were randomised to either brief advice or control treatment. Brief advice consisted of feedback on alcohol and health, written information and an offer of an appointment with an Alcohol Health Worker. Control participants received a leaflet on health and lifestyle. The primary outcome was mean weekly alcohol consumption during the previous 90 days measured 6 months after randomisation. The main secondary outcome was unprotected sex during this period.

**Results:**

Among the 402 randomised to brief advice, 397 (99%) received it. The adjusted mean difference in alcohol consumption at 6 months was −2.33 units per week (95% CI −4.69 to 0.03, p=0.053) among those in the active compared to the control arm of the trial. Unprotected sex was reported by 154 (53%) of those who received brief advice, and 178 (59%) controls (adjusted OR=0.89, 95% CI 0.63 to 1.25, p=0.496). There were no significant differences in costs between study groups at 6 months.

**Conclusions:**

Introduction of universal screening and brief advice for excessive alcohol use among people attending sexual health clinics does not result in clinically important reductions in alcohol consumption or provide a cost-effective use of resources.

**Trial registration number:**

Current Controlled Trials ISRCTN 99963322.

## Introduction

Concerns have been raised about high levels of alcohol consumption among people attending sexual health clinics.^1^ Cross-sectional surveys have repeatedly demonstrated that many people attending sexual health clinics drink above recommended levels.^2^ ^3^ In a consecutive sample of over 500 people attending a sexual health clinic in the south of England, Standerwick and colleagues found that 86% were drinking above recommended limits, and that those drinking excessively were more likely to be diagnosed with a sexually transmitted infection.^4^ Brief advice for excessive alcohol consumption has been shown to be effective across a range of medical settings, and to reduce the likelihood of accidents and injuries,^5^ ^6^ but there is little evidence about its impact when offered to people attending sexual health clinics. The only clinical trial, to date, was conducted by Lane and colleagues in Sydney, Australia.^7^ One hundred and eighty-four people who attended a sexual health clinic and were found to be drinking excessively were randomised to a brief alcohol intervention lasting 5–10 min or to control treatment. Those allocated to the active arm of the trial were more likely to state that they were drinking less 3 months later, but differences in the total score on the Alcohol Use Disorders Identification Test were not found.

The SHEAR (Sexual Health and Excessive Alcohol: Randomised trial) study was set up to examine the clinical and cost-effectiveness of opportunistic brief advice for excessive alcohol use among people who attend sexual health clinics. To achieve this aim we examined whether brief advice reduced subsequent alcohol consumption and altered sexual behaviour 6 months later compared with control treatment, and whether it provided a cost-effective use of resources.

## Methods

The trial was a two-arm, parallel group, assessor-blind, randomised controlled trial. Ethical approval was obtained from West London Research Ethics Committee 3 (10/H0706/29), and the study protocol was registered with Controlled Clinical Trials (ISRCTN 99963322) prior to data collection. The detailed study protocol has been published elsewhere,^8^ and key features are described here.

Study participants were recruited in three sexual health clinics in central and west London. To take part in the study, potential participants had to be aged 19 years or above, be drinking excessively according to the Modified-Single Alcohol Screening Question (M-SASQ),^9^ and be willing to provide written informed consent. According to the M-SASQ, men who drink more than eight standard drinks on one occasion once a month or more, and women who drink more than six standard drinks on one occasion once a month or more are considered to be drinking excessively. We excluded any person who was unable to communicate in English sufficiently to complete baseline questionnaires, anyone who did not have an address or contact telephone number, and anyone who believed they may not be contactable again 6 months later.

### Study procedures

Clinic staff gave all those attending the service a postcard with information about the study. Those who agreed to meet a researcher were given information about the study and asked to provide written informed consent. Following assessment of eligibility, baseline data were collected using a computer-assisted self-completion questionnaire. Participants were then randomised via a remote telephone service provided by an independent Clinical Trials Unit using permuted blocks, stratified by site. Block size was randomly assigned between four and six, with an equal allocation probability between arms. Researchers who collected follow-up data at 6 months were blinded to allocation status. Participants who completed the follow-up interview were offered a £15 honorarium in recognition of their time and any inconvenience related to their involvement in the study.

### Interventions

The SHEAR study had two treatment conditions. Those randomised to control treatment received a general health information leaflet with advice about smoking, alcohol, diet and exercise. In the brief advice group, participants were given feedback from the treating clinician (lasting 2–3 min) which consisted of information about the possible health consequences of excessive alcohol consumption, written information about alcohol and health and an offer of an appointment with an Alcohol Health Worker (AHW). Brief intervention with the AHW lasted up to 30 min and used the ‘FRAMES’ approach which combines active listening and feedback about risks associated with excessive alcohol consumption and emphasises personal responsibility for change.^10^ ^11^ For any participant who was drinking at a harmful or dependent level, the AHW had the option of arranging a follow-up appointment or referring them on to local alcohol services for individual alcohol counselling, detoxification, or other treatments. If the participant was unable to attend an appointment on the day they were seen, then the AHW offered them an appointment at a later date or the option of telephone-based information and advice.

All clinicians who treated study patients received training on delivering brief advice prior to the start of the study. In addition to this, the lead researcher RS spoke to front-line clinicians on the days when recruitment was taking place. She provided support and advice to clinicians, gave feedback on their performance, and checked that brief advice was being delivered in accordance with the trial protocol. All AHWs who took part in the study were experienced practitioners who had undertaken specific training in counselling people who misuse alcohol. They each received regular clinical supervision during the trial and were encouraged to discuss work with trial participants along with other patients they saw during these sessions.

Clinicians delivering brief advice and AHWs were asked to complete a treatment proforma for each person they saw. These proforma were based on ones we used in a previous trial,^12^ and required clinicians to indicate whether they had delivered each of the four components of brief advice and AHWs to record the number and length of session(s), interventions delivered during the session(s) and further information of referrals that were subsequently made. Proforma were examined at the end of the study to examine treatment fidelity.

### Outcome measures

All outcomes were measured 6 months after randomisation and assessed behaviour in the 3 months prior to the date of the assessment. The primary outcome was mean weekly alcohol consumption (measured using the Form 90),^13^ and the main secondary outcome was the proportion of participants who reported any unprotected sex during the previous 3 months. Other secondary outcomes included mean units of alcohol consumed per drinking day, percentage days abstinent (both measured using the Form 90), and whether the participant was drinking excessively (defined as more than eight UK units/64 g of alcohol on one occasion for men, and more than six UK units/48 g for women).^9^ Sexual behaviour outcomes included total number of sexual partners, number of unprotected sexual partners, any incidence of regretted sex, any incidence of unprotected sex after drinking alcohol or while drunk, how long they knew their last sexual partner before they had sex with them, unplanned pregnancy and any new diagnosis of a sexually transmitted infection. These were assessed using questions derived from a previous study.^14^ Finally, we collected data on health-related quality of life (measured using the EuroQol-5 Dimensions scale; EQ-5D),^15^ and health and social care resource use during the past 6 months measured using a modified version of the Adult Service Use Schedule (AD-SUS).^16^ The cost of the brief advice was directly calculated from salaries using a microcosting approach, and national UK unit costs for the year 2010–2011 were applied to medication, hospital contacts and community health and social services.^17^ ^18^

### Statistical methods

The initial sample size calculation was based on identifying differences in mean weekly alcohol consumption as found in our previous trial of brief advice in an emergency department^12^ and suggested a minimum of 160 per arm.^8^ However, in the first few months of the trial the rate of recruitment was higher than expected and the sample size was therefore increased to provide additional power to test the primary and main secondary outcomes. The final sample size was based on a practical size of 380 per arm (760 in total). If the intervention reduced the proportion of participants who had unprotected sex from 65% to 50%, the power to detect a significant difference would be above 90%, assuming 25% drop out, and a clustering design effect of 1.15.

The statistical methods and trial design were specified a priori in a protocol paper^8^ and in a further detailed Statistical Analysis Plan.^19^ All analysis was performed in STATA (V.12). The primary outcome, mean weekly alcohol consumption, was compared between the randomised groups using random-effects linear regression, adjusted for age, sex and harmful alcohol use at baseline. The random-effects model takes into account clustering by sexual health clinic and, in the intervention arm, by treating clinician.^20^ Despite the skewed distribution of the outcome data, we used ordinary parametric models, which enables inference to be made about the arithmetic mean and are sufficiently robust to skewed outcome in a large sample.^21^ Robustness of the result was assessed by various sensitivity analyses, including non-parametric bootstrapping and non-hierarchical linear models. The main secondary outcome, proportion of participants reporting any unprotected sex, was analysed using random-effects logistic regression, adjusted for age, gender, and unprotected sex in the previous 6 months at baseline. Other secondary outcomes were analysed by linear, logistic or negative binomial regression. For rare outcomes, exact tests were used, and CIs calculated using mid-p method.^22^ All analyses were carried out according to the allocated randomisation arm, and two-sided p values were considered significant when below 0.05.

Patients with missing data were excluded in primary ‘complete case’ analyses, with multiple imputation using chained equations performed as a sensitivity analysis.^23^ Outcomes, covariates, predictors of outcomes and predictors of missingness were included in the imputation model, with both arms imputed separately in order to allow for interactions. A range of Missing Not at Random mechanisms were then considered in further sensitivity analyses to assess the robustness of the primary results.^24^

The economic evaluation took a NHS/Personal Social Service perspective and had a 6-month time horizon. Standard parametric tests were used for the analysis of cost data, as recommended,^21^ with the robustness of the test confirmed using non-parametric bootstrapping.^25^ Effectiveness was assessed in terms of health-related Quality Adjusted Life Years (QALY) derived from the EQ-5D.^26^ The cost-effectiveness of the brief advice was assessed through the generation of cost-effectiveness acceptability curves (CEACs), which present the probability that the advice is cost-effective for different values a decision maker might be willing to pay for an improvement in outcome.^27^

## Results

**Figure 1:**
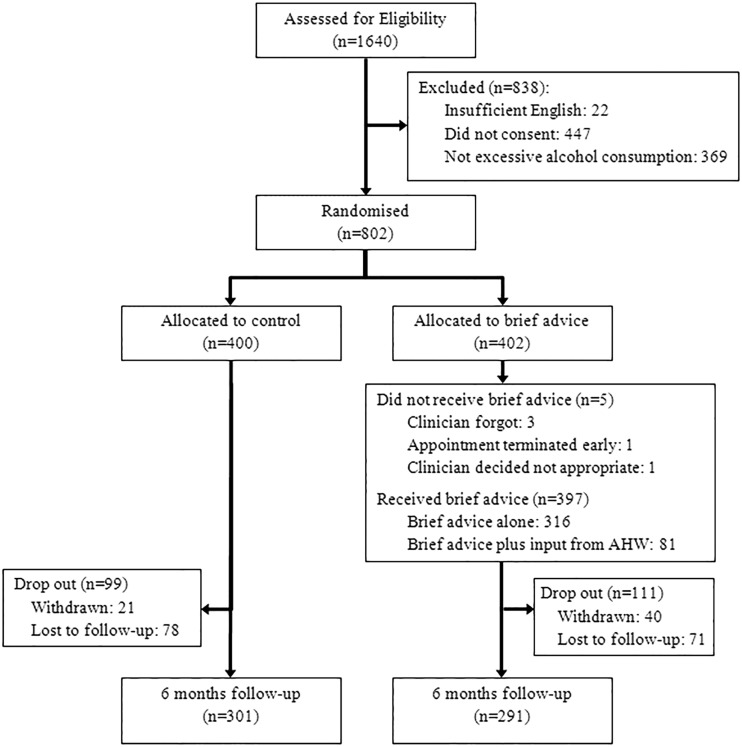
Study flow-chart.

Of 1649 people who were assessed for the study between August 2010 and May 2012, 802 (49%) met our inclusion criteria. Recruitment stopped at this point as the revised target sample size had been exceeded and a decision was made to stop recruitment in a trial management meeting. Of those randomised, 592 (74%) were followed-up at 6 months. Reasons for non-participation and attrition are presented in figure 1, and baseline characteristics of participants are presented in table 1. Participants had a median age of 27 years (IQR=24 to 30), and 432 (54%) were female. All but five (1.2%) of the 402 participants who were randomised to the active arm of the trial received brief advice from the treating clinician. Among 370 in the active arm of the trial who were asked whether there was a link between their alcohol use and their attendance at the clinic, 70 (19%) said there was.

**Table 1:** Sociodemographic and clinical characteristics of study participants at baseline

Variable	Control N=400	Brief advice N=402	Total N=802
Clinic (%)			
Hospital Site 1	247 (61.8)	248 (61.7)	495 (61.7)
Hospital Site 2	103 (25.8)	103 (25.6)	206 (25.7)
Hospital Site 3	50 (12.5)	51 (12.7)	101 (12.6)
Gender (%)			
Male	183 (45.8)	187 (46.5)	370 (46.1)
Female	217 (54.3)	215 (53.5)	432 (53.9)
Age (median, IQR) (N=801)	26.8 (23.4–30.4)	26.3 (23.7–30.4)	26.7 (23.6–30.4)
Ethnicity (N=799) (%)			
White	309 (77.6)	309 (77.1)	618 (77.3)
Black/ Mixed	52 (13.1)	52 (13.0)	104 (13.0)
Asian/ Mixed	13 (3.3)	16 (4.0)	29 (3.6)
Other	24 (6.0)	24 (6.0)	48 (6.0)
Sexual orientation (N=801) (%)			
Heterosexual	361 (90.5)	325 (80.8)	686 (85.6)
Homosexual	34 (8.5)	59 (14.7)	93 (11.6)
Bisexual	4 (1.0)	18 (4.5)	22 (2.7)
Smoking status (N=801) (%)			
No	228 (57.1)	228 (56.7)	456 (56.9)
Yes	171 (42.9)	174 (43.3)	345 (43.1)
Reason for presentation (N=788) (%)			
Sexual health check only	166 (42.3)	175 (44.2)	341 (43.3)
Symptoms	188 (48.0)	185 (46.7)	373 (47.3)
Emergency Contraception	6 (1.5)	8 (2.0)	14 (1.8)
Further Treatment/ Vaccination	20 (5.1)	17 (4.3)	37 (4.7)
Other	12 (3.1)	11 (2.8)	23 (2.9)
Drinking 6+/8+ units in one session (N=801) (%)			
Monthly	141 (35.3)	153 (38.1)	294 (36.7)
Weekly	253 (63.4)	242 (60.2)	495 (61.8)
Daily	5 (1.3)	7 (1.7)	12 (1.5)
Had unprotected sex in the last six months (N=801) (%)			
No	45 (11.3)	78 (19.4)	123 (15.4)
Yes	354 (88.7)	324 (80.6)	678 (84.6)
Number of unprotected sexual partners in the last six months (N=801)	Mean 1.7 (SD 1.6)	Mean 1.4 (SD 1.3)	Mean 1.6 (SD 1.4)
Health related quality of life (EQ-5D) (N=801)	0.892 (SD 0.170)	0.893 (SD 0.166)	0.892 (SD 0.168)

Data from treatment proforma showed that, of the 402 randomised to brief intervention, 397 (99%) received brief feedback that alcohol use at that level has the potential to harm health, 370 (92%) were asked whether there was a link between alcohol use and attendance at the clinic, 397 (99%) were given a leaflet on alcohol and health, of whom 372 (92.5%) took the leaflet, and 397 (99%) were offered an appointment with an AHW.

Of the 397 offered an appointment with an AHW, 81 (20%) received input either by phone (N=48) or in a face-to-face meeting (N=33). Follow-up researchers reported nine occasions when they became aware of a participants’ allocation status, all were from the intervention arm.

Primary and secondary outcomes at the 6 month follow-up are described in table 2. At 6 month follow-up, participants in the active treatment arm of the trial were drinking a mean of 18.1 units per week compared to 20.3 units among controls; adjusted mean difference in alcohol consumption=−2.33 units per week (95% CI −4.69 to 0.03, p=0.053). Unprotected sex was reported by 154 (53%) of those randomised to brief advice and by 178 (59%) of controls (adjusted OR=0.89, 95% CI 0.63 to 1.25, p=0.496). Sensitivity analyses regarding statistical model used and missing data, gave similar findings of a small difference around statistical significance for the primary outcome.

**Table 2:** Outcomes at six month follow-up by trial arm

Outcome over the last three months	Control N=301	Brief advice N=291	Coefficient/OR* 95% CI
Weekly alcohol consumption (units)			
Mean (SD)	20.3 (16.6)	18.1 (15.6)	−2.33
Median (IQR)	15.7 (8.3–29.9)	14.1 (6.5–25.1)	−4.69 to 0.03
Had unprotected sex (%)			
No	123 (40.9)	137 (47.1)	0.89
Yes	178 (59.1)	154 (52.9)	0.63 to 1.25
Average units on drinking days			
Mean (SD)	10.4 (5.8)	9.3 (5.3)	−1.13
Median (IQR)	9.4 (6.5–13.4)	8.6 (5.6–11.4)	−1.96 to −0.29
Proportion of days abstinent			
Mean, (SD)	70.7 (22.6)	70.9 (22.1)	0.20
Median (IQR)	75.6 (62.2–87.8)	75.6 (58.9–87.8)	−3.03 to 3.44
Drinking excessively (M-SASQ) (%)			
No	55 (18.3)	70 (24.1)	0.70
Yes	246 (81.7)	221 (75.9)	0.46 to 1.05
Number of sexual partners			
Mean (SD)	1.9 (2.9)	1.6 (2.2)	−0.13
Median (IQR)	1 (1–2)	1 (1–2)	−0.29 to 0.02
Number of unprotected partners			
Mean (SD)	0.8 (1.1)	0.6 (0.8)	−0.11
Median (IQR)	1 (0–1)	1 (0–1)	−0.31 to 0.08
Occurrence of regretted sex (%)			
No	273 (90.7)	263 (90.4)	1.05
Yes	28 (9.3)	28 (9.6)	0.60 to 1.84
Unprotected sex after drinking (%)			
No	165 (54.8)	183 (62.9)	0.79
Yes	136 (45.2)	108 (37.1)	0.56 to 1.11
Unprotected sex after feeling drunk (%)			
No	245 (81.4)	234 (80.4)	1.15
Yes	56 (18.6)	57 (19.6)	0.76 to 1.75
Unplanned pregnancy (n=316)			
No	160 (98.8)	152 (98.7)	1.05
Yes	2 (1.2)	2 (1.3)	0.16 to 6.79
New diagnosis of sexually transmitted infection			
No	287 (95.3)	283 (97.3)	0.58
Yes	14 (4.7)	8 (2.7)	0.25 to 1.384
Health related quality of life (SD)	0.922 (0.144)	0.910 (0.150)	−0.013 −0.037 to 0.109

*Coefficient/OR from linear, logistic or negative binomial (number of sexual partners) regression for difference between arms, adjusted for age, gender, clinic and corresponding variable at baseline. Except for unplanned pregnancy and STI diagnosis where OR are unadjusted, CI mid-p estimates, and p value from Fisher's exact tests. M-SASQ, Modified-Single Alcohol Screening Question .

The active intervention cost £12.57 on average (SD 6.59) which was a relatively small addition to total costs (mean 6-month costs £319) (table 3). The control group cost £8 less on average (mean 6-month cost £311). There were no significant differences in costs (8.41; 95% CI −98.56 to 115.37, p=0.879) or QALYs, which were 0.007 lower in among those allocated to brief advice (95% CI −0.017 to 0.003, p=0.190). The cost-effectiveness acceptability curve (see figure S2—web only) demonstrates that there is no evidence that brief advice is cost-effective at any willingness to pay values for a QALY.

**Table 3:** Mean cost (£) and Quality Adjusted Life Years (QALYs) per participant over 6-month follow-up

	Control n=301		Brief advice n=291		Difference	95% CI
	Mean	(SD)	Mean	(SD)		
Brief alcohol advice	0	(0)	12.57	(6.59)		
Outpatient appointments in sexual health clinic	23.97	(55.16)	25.55	(51.75)		
All other hospital services	152.35	(446.7)	133.13	(359.05)		
Community health and social services	74.59	(256.89)	60.73	(165.01)		
Medication	59.96	(235.87)	87.3	(404.71)		
Total cost	310.87	(681.12)	319.28	(662.69)	8.41	−98 to 115.37
QALY	0.457	(0.063)	0.450	(0.066)	−0.007	−0.0174 to 0.003

## Discussion

Data from this randomised trial of brief advice for excessive alcohol use among people attending sexual health clinics suggests that there is little, if any, difference in alcohol consumption between those who are and are not offered this advice. At 6 months, people randomly allocated to receive brief advice were consuming a mean of 2.3 units (18.4 g) of alcohol less per week than those randomised to the control group. In keeping with recommendations for interpreting the results of borderline statistically significant results^28^ we base this conclusion on the clinical significance of the changes we found rather than on the probability of the difference being the result of chance. While a statistically significant difference in number of units of alcohol per drinking day was observed, the scale of the difference (1.1 units/8.8 g), is unlikely to be clinically important. Significant differences in sexual health outcomes were not found between the groups, though we cannot rule out the possibility that brief advice could be associated with small but clinically important changes in sexual health outcomes. At under £13 per participant, brief advice was inexpensive. However, when other costs and QALY outcomes were taken into account we found that that brief advice is very unlikely to provide a cost-effective use of resources.

### Strengths and weaknesses

The study was sufficiently powered to examine clinically important differences in the primary outcome. We tested an intervention that was delivered by front-line clinicians and could be rolled out to other clinics. Limitations of the study were that all recruitment took place in sexual health clinics in London and, through limiting exposure of control participants to questions on alcohol, we had only a small amount of information about alcohol consumption at baseline. However, the little information we did collect suggests that the groups were well balanced. We were unable to collect follow-up data from a quarter of the study sample. Excluding these participants from the main analysis could have biased the intervention effect estimate. However, sensitivity analyses demonstrated that this was unlikely to significantly affect the conclusions of the trial.

### Comparison with other studies

The only previous trial of an intervention for excessive alcohol use among people attending sexual health clinics reported that people offered active intervention believed they were drinking less 3 months later.^7^ However, mean scores on the primary outcome measure, Alcohol Use Disorders Identification Test, were very similar (11.5 in controls and 10.7 among those offered brief intervention). This study was not large enough to detect clinically important differences in alcohol consumption between study groups. The results of the SHEAR study provide stronger evidence that interventions aimed at reducing alcohol consumption among people attending sexual health clinics may have very limited, if any, impact.

This study confirmed high levels of excessive alcohol consumption among people attending sexual health clinics. While most people were drinking above recommended limits median weekly alcohol consumption was lower than reported in previous studies.^5^ ^12^ There is some evidence that brief advice is less effective among people who have lower levels of alcohol consumption,^5^ and this may be one of the reasons we did not find clinically important differences in alcohol consumption between those who did and did not receive brief advice.

It is of note that far fewer participants in this study said that they thought there was a link between their alcohol consumption and the reason for their presentation to the sexual health clinic than has been reported in studies in emergency departments (19% in this study compared to 51% in the latter).^29^ While people attending sexual health clinics may want to achieve better sexual health, attempts to reduce alcohol consumption may not be seen by them as a necessary means of trying to achieve this aim.

A number of clinical trials of brief intervention have been conducted in different outpatient clinics.^30^ While patients treated in specialist oral and maxillofacial clinics may benefit from brief intervention for excessive alcohol use, those treated in general medical clinics may not. The findings of the SHEAR trial are in keeping with those from studies conducted in general medical clinics, and challenge current national guidelines in England and Wales that healthcare professionals should routinely carry out alcohol screening when promoting sexual health.^31^

## Conclusions

The approach we used in this trial to screen and deliver brief advice for excessive alcohol consumption among people attending sexual health clinics did not result in clinically important reductions in alcohol consumption or provide a cost-effective use of resources. Alternative approaches to supporting people who drink excessive alcohol should be developed, tested and found to be effective before they are introduced into sexual health clinics.

**Key messages**

- Most people attending sexual health clinics drink alcohol above recommended levels. Those who do so are more likely to be diagnosed with sexually transmitted infections.
- Brief advice for excessive alcohol consumption is effective in a range of medical settings, but its impact in sexual health clinics is not known.
- Most people who attend sexual health clinics and drink excessively are willing to accept brief advice, but few take up the offer of additional interventions.
- Brief alcohol advice, as delivered in this trial, did not lead to clinically significant reductions in alcohol use or changes in sexual behaviour.

## Supplementary Material

Web supplement
