# Supplementary material for: The clinical and cost-effectiveness of brief advice for excessive alcohol consumption among people attending sexual health clinics: a randomised controlled trial
Source: Sex Transm Infect. 2014 Jun 16;91(1):37–43. doi: 10.1136/sextrans-2014-051561 (PMC4316933; doi:10.1136/sextrans-2014-051561)

FIGURE 2 **(Web Only)** Cost-effectiveness acceptability curve showing probability that brief advice is cost-effective compared with usual care for different values of willingness to pay per QALY.

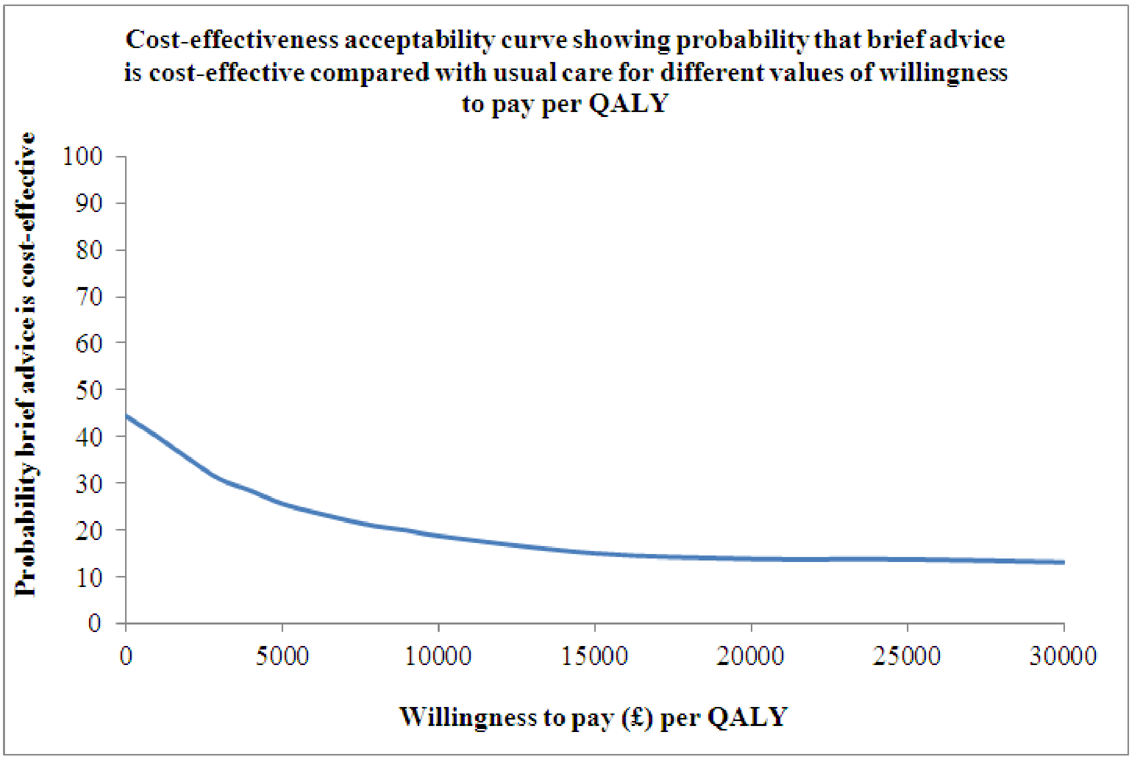

Supplement: Web supplement [file sextrans-2014-051561-s1.pdf]
